# A251 GUT-SPECIFIC DOWNREGULATION OF INTERFERON-LAMBDA RESPONSES OCCURS IN INFLAMMATORY BOWEL DISEASES

**DOI:** 10.1093/jcag/gwad061.251

**Published:** 2024-02-14

**Authors:** O Ogungbola, R Mahmood, J Ouyang, V Vu, N Nguyen, X Liu, K Bittorf, S Lamb, W El-Matary, C Bernstein, E Wine, L D Tyrrell, H K Armstrong, D Santer

**Affiliations:** Immunology, University of Manitoba Faculty of Health Sciences, Winnipeg, MB, Canada; University of Manitoba Department of Internal Medicine, Winnipeg, MB, Canada; Immunology, University of Manitoba Faculty of Health Sciences, Winnipeg, MB, Canada; Immunology, University of Manitoba Faculty of Health Sciences, Winnipeg, MB, Canada; Immunology, University of Manitoba Faculty of Health Sciences, Winnipeg, MB, Canada; Immunology, University of Manitoba Faculty of Health Sciences, Winnipeg, MB, Canada; Li Ka Shing Institute of Virology and Medical Microbiology and Immunology, University of Alberta, Edmonton, AB, Canada; Li Ka Shing Institute of Virology and Medical Microbiology and Immunology, University of Alberta, Edmonton, AB, Canada; University of Manitoba Department of Pediatrics and Child Health, Winnipeg, MB, Canada; University of Manitoba Department of Internal Medicine, Winnipeg, MB, Canada; Center of Excellence for Gastrointestinal Inflammation and Immunity Research and Department of Pediatrics, University of Alberta, Edmonton, AB, Canada; Li Ka Shing Institute of Virology and Medical Microbiology and Immunology, University of Alberta, Edmonton, AB, Canada; University of Manitoba Department of Internal Medicine, Winnipeg, MB, Canada; Immunology, University of Manitoba Faculty of Health Sciences, Winnipeg, MB, Canada

## Abstract

**Background:**

Inflammatory bowel diseases (IBD) affect 1:140 Canadians, resulting in significant inflammation and gut damage. Unlike type I and II interferons (IFNs), the most recently discovered IFNs, interferon-lambdas (IFN-λs), uniquely downregulate gut inflammation and promote gut healing in mouse models. However, it remains unknown if the important anti-inflammatory IFN-λ signaling pathways induced downstream of the IFN-λR1/IL-10RB heterodimeric receptor are altered in IBD.

**Aims:**

To compare intestinal and peripheral IFN-λR levels and responses in people with active or remission IBD and those without IBD. We hypothesized that intestinal IFN-λR1 levels and downstream activities are decreased in people living with IBD, which could contribute to IBD pathology (gut inflammation and mucosal damage).

**Methods:**

During routine colonoscopy, uninflamed biopsy samples were obtained from the terminal ileum and sigmoid colon. IFN-λR1 levels were quantified in intestinal biopsy and blood samples from persons with or without IBD (n=12 adults and n=12 pediatric) by immunohistochemistry and flow cytometry. Fresh intestinal biopsies were also cultured *ex vivo* for 24 hr in media +/- IFN-λ3 and gene expression was quantified by RT-qPCR.

**Results:**

In IBD gut tissues, a significant decrease in IFN-λR1+ cells was more prominent in IBD patients with active disease (pampersand:003C0.01, 30-50% reduction). The decrease was more prominent in IBD patients with active disease. Consequently, there was a significantly lower IFN-λ-driven upregulation of genes such as IFIT1 and MX1 in IBD as compared to non-IBD tissues (pampersand:003C0.05, 4-7-fold reduction). Interestingly, IFN-λR1 levels were not decreased in peripheral blood immune cells, indicating the dysregulation of IFN-λR responses is likely gut-specific.

**Conclusions:**

Our findings demonstrate that people living with IBD have dysregulated IFN-λ responses in the gut which could lead to the lower induction of key anti-inflammatory pathways. This work supports the further study of mechanisms regulating the IFN-λ system to develop strategies to restore and promote IFN-λ responses as a novel potential future IBD therapy.

**Funding Agencies:**

University of Manitoba, Weston Family Foundation, The Children's Hospital Research Institute of Manitoba.

